# Clinical etiology of myiasis in ENT: a reterograde period - interval study

**DOI:** 10.1016/S1808-8694(15)30651-0

**Published:** 2015-10-19

**Authors:** Shitij Arora, J.K. Sharma, S.K. Pippal, Yatin Sethi, Abhinav Yadav

**Affiliations:** 1 Resident (Gandhi Medical College, Bhopal, India); 2 Professor (Head Of Deptt, Gandhi Medical College, Bhopal, India); 3 Assoc Professor (Deptt Of Ent, Gandhi Medical College Bhopal); 4 Resident (Gandhi Medical College); 5 Resident (Gandhi Medical College,)

**Keywords:** laryngeal, myiasis, nasal, tracheostomy

## Abstract

Myiasis in ENT, once a deadly disease still presents as a significant outdoor problem, though advances in management including supportive therapy has led to early healing with significant reduction in bed occupancy rate.

**Aims:**

To assess the clinico etiology, relationship of myiasis to habit and habitat of patients and to assess the changes in age, seasonal, socioeconomic incidence, nasal bacterial flora and usefulness of certain commonly done tests with reference to a gap of 25 years.

**Materials & methods:**

The presenting study was conducted on 80 patients selected over a period of two time intervals; first 40 cases were chosen from 1979 to 1980 and next 40 over 2003 to 2004. Cases were studied in a retrograde manner and data tabulated.

**Results & Conclusions:**

Atrophic turbinates was the commonest pathological finding in nose in 30% of cases. Significant change seen was in the age group 51 and above with a rise of 30%. Mode during 2003-04 was 60 years. Incidence of palatal perforation dropped from 17.88 to 2.5%. Klebsiella emerged as a significant contributor to the nasal microbial flora. VDRL and split skin smear showed poor etiological association for the diseases.

## INTRODUCTION

Nostalgia - Myiasis in ENT can be traced back to ancient Hindu mythological works, in those days it was supposed to be due to commision of sins and the wrath of saintly persons on them. In new world Soares 'd' Souza 1587 reported just one case of cutaneous myiasis.

The tem myiasis is of recent origin and Rev F.W. Hope coined the term in 1840 (Previously it was called scholechiasis). Stecle in 1897 proposed that the presence of fly in nasal spaces causes myiasis.

Castellani and Chalmer's in 1919 described the condition of nasal myiasis, known as Peenash in India, due to chrysomia (formerly pycnosoma).

### Classification of Myiasis

Bishop et al. 1926 classified the maggots as tissue destroying, subdermal, i nfesting gastrointestinal/ urogenital tract, ear nose & throat and blood sucking types.

### Present classification (Sahay et al, 1958)

(a) Specific - obligate parasite where maggot is unable to complete its life cycle without appropriate host.
(b) Semi specific - Species are adaptable to environment, pH, temperature.
(c) Accidental - Invasion of facultative parasites.

Once a deadly disease, advances in management including supportive therapy has led to the early healing with significant reduction in the hospital stay. A disease of the people belonging to low socioeconomic strata previously stated to be prevalent during a period post rains is now fast becoming a perennial disease. Earlier it was treated with crystalline penicillin and removal of maggots. Crystalline penicillin was used on a rationale based on the reports that Staphylococcus aureus was the most common cultured organism from nasal cavities in affected patients. The patients were subjected to tests to identify the causes of atrophic rhinitis such as syphilis and leprosy.

The aim of this study is to assess the clinico-etiology, the relation of myiasis to habit and habitual of patients with special reference to changing trends.

### Surgical Maggots:

Observed since was the certain infected wounds exposed to maggots healed rapidly (Baer – 1931).

Lingstone Prince (1932) pointed out on the therapeutic role of maggots in long standing cases of CSOM.

All though extremely rare in the western world this disease is not infrequent in the dry, warm, tropical climate seem in south-east Asian region.

## MATERIALS AND METHODS

A retrospective study was conducted on 80 patients from department of ENT, Gandhi Medical College and Associated Hamidia Hospital, Bhopal for associated work out. Ethical committee approval was sought and the permission order no. 851076/mc/7/07 dated 16.04.07 is quoted.

Patients were divided into 2 groups.

Group I - Dated 1979-1980 – 40 patients

Group II – 2003-2004 – 40 patients.

The patients in this study were then categorized according to

• Age
• Sex
• Presenting complaints
• Finding on initial examination
• Regional distribution
• Seasonal variation
• Occupation, Socioeconomic variation, literacy.
• Bacteriological study of nasal cavity / Secretion.

The following pattern of examination and investigation was adopted for each case in present study.

### History of Presenting Illness:

1. History regarding - Nasal disease, epistaxis, pain, swelling of nose, passing worms from nose.
2. History of discharge, bleeding pain and passing worms from ears.
3. Any history of passing worms from mouth, difficulty in swallowing toothache, swelling of gums, ulcer in mouth, vomiting.
4. Tracheostomy wound condition.

### History of Past Illness:

Any history which could be a predisposing factor for myiasis in ENT such as crust formation in nose / ozaena, loss of smell, bleeding, foul smelling discharge from ear.

### Personal History:

Detailed inquiry about social status, condition of surrounding, sanitation.

### Clinical Examination:

(a) General examination: A brief general examination was conducted to assess the nutritional status and built of patient, degree of dehydration, anemia or any associated disease.
(b) Systemic examination: Examination of CNS Respiratory system, CVS with regard of any abnormality.
(c) ENT Examination

Ears:

(a) External ear (Pinna) was examined for any deformity, swelling, infected wound, presence of maggots.
(b) EAC - Discharge - Purulent / mucopurulent / maggots / bleeding.
(c) Tympanic membrane - Looked for any congestion, perforation mastoid region was looked for any swelling / furuncle.

Nose: External deformity, deviation / perforation of septum, nasal discharge, presence of crust, atrophy of nasal mucosa turbinates, presence of maggots.

Throat: In examination of throat teeth and gums were first inspected for dental caries / swelling of gums, palate examined for any bulging perforation. Tonsils, tonsillar fauces, PPW were examined for any congestion / presence of maggots. Posterior rhinoscopy, indirect laryngoscopy was done in all cases and findings were noted.

Investigation: Following routine and special investigations were carried out -

1. Blood examination, Hb%, degree of anaemia.
2. Urine - Sugar, Albumin.
3. Bacteriological, examination - Swab were taken on day of admission before local medicine was instilled in involved region. Material for culture was obtained from ear, nose, throat as the case may be using a sterile thin cotton wool swab especially prepared and autoclaved and care was taken to immediately secure the swab in autoclaved test tube, swab were sent to microbiology laboratory for analysis.
4. For cases in 2003-2004 - VDRL and skin smear for lepra bacill.

Collection of Maggots: Prior to collection of maggots from nose and nasal wash with turpentine solution along with NaCl, Sodium bicarbonate, Sodium biborate in the specific ratio.

Maggots were then picked up manually from nasal cavity with the help of nasal speculum and Tilley's forceps.

In aural myiasis antibiotic / steroid eardrops was instilled and maggots removed with Tilleys forceps.

In pharyngeal myiasis irrigation was done with one nostril closed to allow the fluid run through mouth.

## RESULTS

The present study comprises a clinico-etiological study of 40 cases of myiasis in ENT dated 1979-1980 and 40 cases in period dated 2003-2004 admitted in Hamidia Hospital, Bhopal.

The study was carried out with a view to establish the factors responsible for the condition and changing trends with reference to a gap of 25 years.

he patients were also scrutinized for their socioeconomical status, religion, habitat, personal hygiene other associated diseases. Following observations have been made during current study.

**Chart 1:**
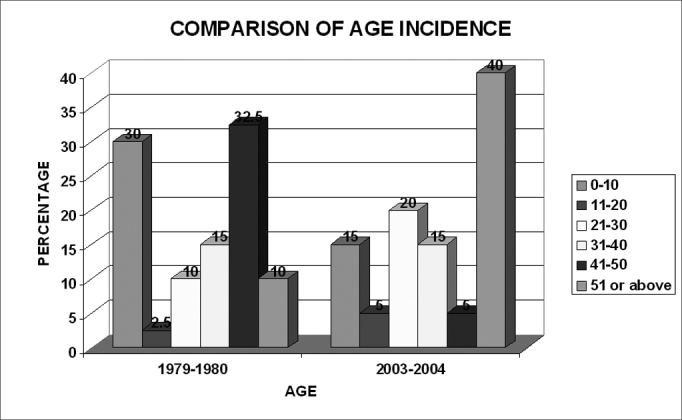
Comparison of age incidence between the two periods

**Table 1:** Sex Incidence.

Sex	No. of Patients (%)	
	1979– 1980	2003– 2004
Male	13 (32,5%)	18 (45%)
Female	27 (67,5%)	22 (55%)

**Table 2:** Socio Economic Variation: Arbitrary Selection of Income.

Class	Rs/month	No. of Patients (%))	
		1979-80	2003-04
Poor	<300	34(85%)	38(95%
Middle	300-600	05(12,5%)	02(5%)
High	>600	01(2,5%)	–

**Table 3:** LITERACY (Assessed by level of Primary Education).

Literacy Status	No. of Patients (%))	
	1979-80	2003-04
Literate	03(7,5%)	04(10%)
Illiterate	37(92,5%)	36(90%)
Total	40	40

**Tabela 4:** Occupation.

Occupation	No. of Patients (%))	
	1979-80	2003-04
Housewife	27(67,5%)	24(60%)
Labourer	07(17,5%)	10(25%)
Farmer	03(7,5%)	02(5%)
Student	01(2,5%)	03(7,5%)
Painter	01(2,5%)	0
Bussinessman	01(2,5%)	01(2,5%)
TOTAL	40	40

**Table 5:** Seasonal Variation.

Season	No. of Patients (%))	
	1979-80	2003-04
Jan- March	0	04(10%)
April- June	12 (30%)	0
July- Sep	01(2,5%)	06(15%)
Oct- Dec	27(67,5%)	30(75%)
Total	40	40

**Table 6:** Bacterial Flora.

Organism	No. of Patients (%))	
	1979-80	2003-04
Staphlyococcus aureus	75%	90%
E. coli	25%	2%
Klebsiella	–	8%

**Table 7:** Specific Site.

Site	No. of Patients (%))	
	1979-80	2003-04
Nasal	70%	75%
Aural	27.5%	17.5%
Pharyngeal	2.5%	2.5%
Outros	–	5%

**Chart 2:**
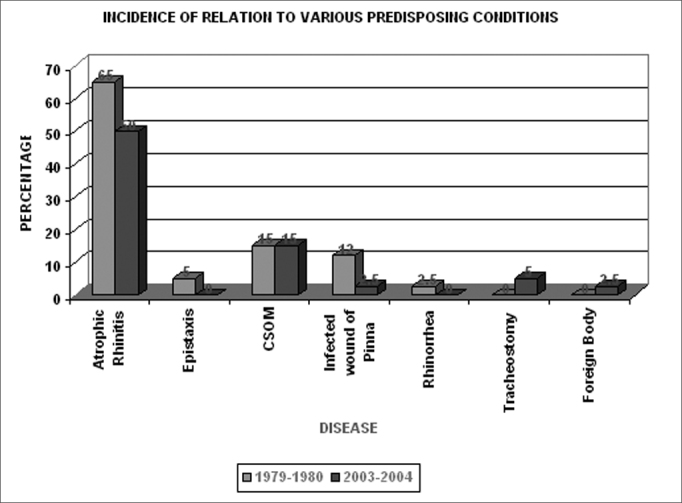
The various predisposing conditions found with myiasis

**Chart 3:**
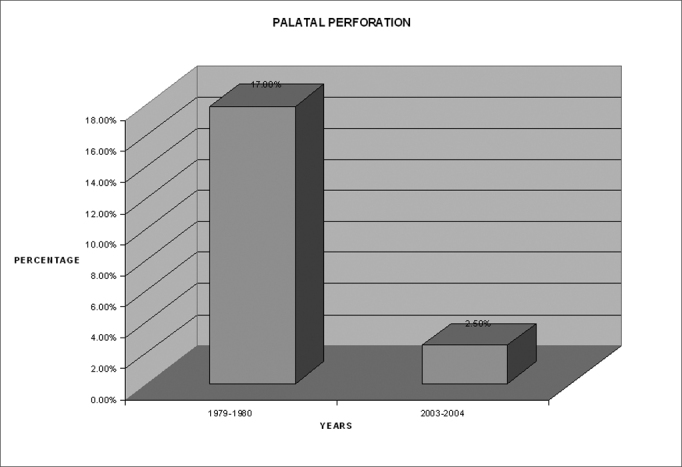
Comparison of incidence of palatal perforation between the two periods

### Incidence of VDRL positivity in the present period Interval

2003-2004

Titre = 1:32 dilation

Cases studied – 40

VDRL - negative all 40

**Figure 1:**
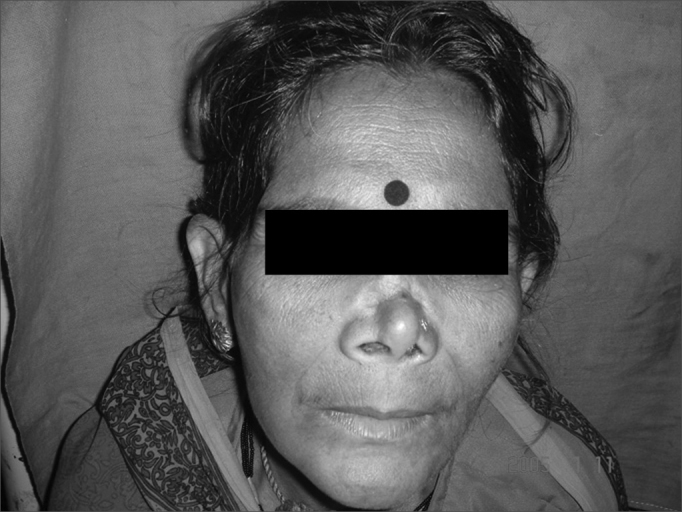
patient with nasal myiasis showing depressed dorsum

**Figure 2:**
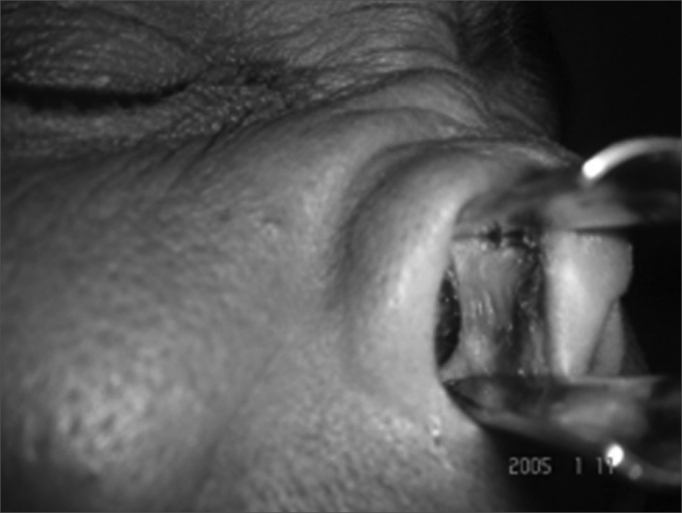
The same patient with septal perforation

### SPLIT SKIN SMEAR(for lepra bacilli)

Cases = 40

Positive = 1 (patient had thickened ulnar nerves)

Percentage = 2.5

**Figure 3:**
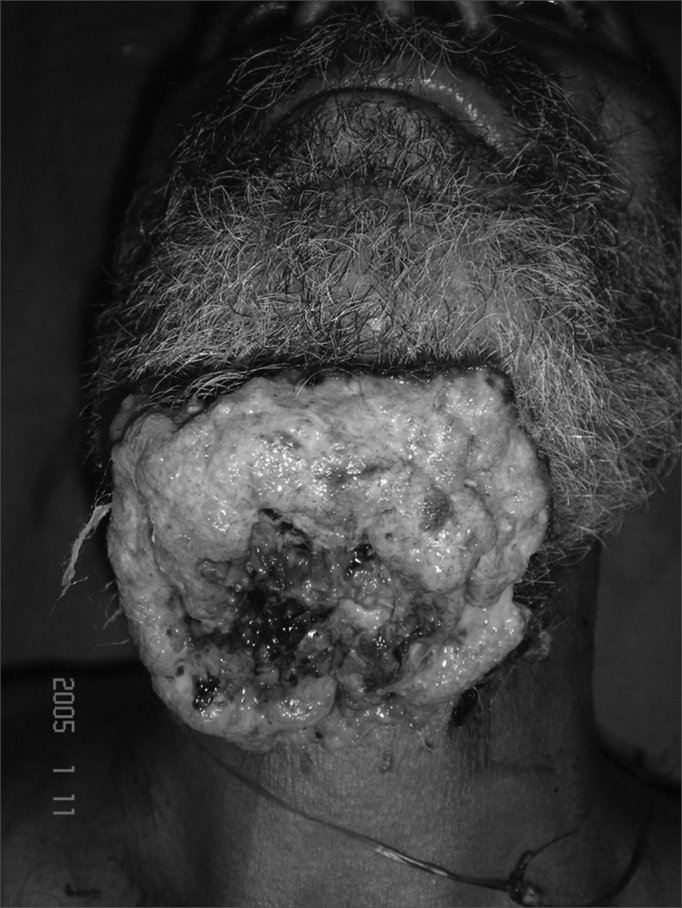
Patient with myiasis of a large growth of the floor of mouth invading the skin

**Figure 4:**
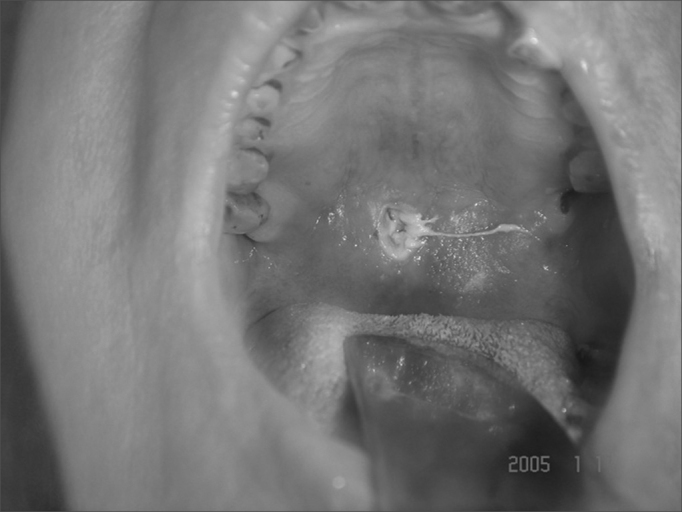
A patient with palatal perforation secondary to nasal myiasis

## DISCUSSION

The exact mode of evolution of myiasis in not known. There are two schools of thought -

1. Rao – 1929 - This lay eggs directly in side cavity.
2. Sood - Kakkar – 1975 - This lay eggs in vicinity of aural / nasal cavity when patient is sleeping.

There is possibility of eggs being transferred into nasal / ear cavities by patients own finger also due to poor hygienic conditions. Out of split skin smear done on 40 patients in 2003-2004 one had positive lepra bacilli and he also had thickened palpable cutaneous nerves.

The explanation of nose being affected much more is due to -

• Easy accessibility
• Wider space.
• Relatively less sensitive mucosa than ear.

High incidence of aural myiasis below 10 years of age is due to

• Improper care.
• Superimposed unhygienic living specially in lower socio-economic groups.

Laryngeal tissue involved when exposed.

Rare otherwise due to

• Inaccessibility
• High sensitivity.
• Cough reflex

### Age Incidence

30% of cases seen during 1979-1980 amongst between age group 0-10 dropped to 15 by period.

2003-2004

Due to -

• Improved pediatric care system.
• Hygienic level

But a significant change seen was in the age group 51 - above with the percent affected cases in 2003-2004 raising sharply to 40 from the 10 in 1979-1980.

Some introspect needed in this regard.

Mode 2003-2004 – 60 years.

### Sex Incidence

Gap between male and female has narrowed.

### Seasonal Variation

Oct - Dec was the most commonly observed period. But a significant number of cases seen in Jan - March (10%), so it is fast becoming a Perennial Disease

Other Statistics

Sood & Kakkar: Sept. - Nov.

Sahay (Patna): May - June

Bhatia: Aug. - Nov.

### Palatal Perforation

Rate dropped from 12.5% to 2.5%. Probably due to effective manual removal of nasal maggots.

### BaCterial study

Klebsiella is emerging as a common pathogen.

## CONCLUSION

1. Myiasis constitutes a significant number of cases attending ENT outdoor.
2. Clinico-etiological study was carried out which was compared between two period intervals
3. Both periods had the disease prevalent among poor people living in unhygienic condition.
4. Winter was the most common season for myiasis with maximal cases occurring during Oct. - Dec. period. Though a significant rise was observed during the period of Jan. to Mar.
5. The disease was seen mostly in elderly and was least common in children.
6. VDRL and investigations for lepra bacilli should be limited to clinically
  Symptomatic patients and not subjecting them to an extra investigation.
7. Klebsiella emerged as a new pathogen in diseased individuals of the later period possibly explaining the importance of cephalosporins in the management.
